# Plant and animal organelles in cell death

**DOI:** 10.18632/oncotarget.4386

**Published:** 2015-06-08

**Authors:** Hong Yu, Jiayang Li

**Affiliations:** State Key Laboratory of Plant Genomics and National Center for Plant Gene Research (Beijing), Institute of Genetics and Developmental Biology, Chinese Academy of Sciences, Beijing, China

Programmed cell death (PCD) is a finely regulated and genetically controlled process which plays crucial roles in various biological courses. In human, inappropriate PCD could lead to various diseases including neurodegenerative diseases, ischemic damage, autoimmune disorders, and many types of cancers [1]. Over decades, great efforts have been made in identifying key cell death genes in various model animals and more distinct types of PCD have been observed. These genes show evolutionary conservation among *C. elegans, Drosophila, Rattus* and *Homo sapiens* [2]. At the subcellular level, mitochondria act as the key organelle in PCD and display a central role in both induction and execution of PCD [3]. Reactive oxygen species (ROS), which mainly produced by mitochondria in animal, could trigger the initiation of apoptosis through both the extrinsic and intrinsic pathways. Recently, a novel type of vertebrate specific PCD termed necroptosis has been found showing a pivotal role in inflammation and immunity, in which the elevation of ROS levels is recognized as a hallmark and may be one of the main causes [2].

In plant, the knowledge of PCD is relatively obscure. Similar morphological and biochemical features of PCD are observed between animal and plant, including cell shrinkage, chromatin condensation, nuclear fragmentation, cytochrome complex release, endonuclease release etc. [4]. Various caspase inhibitors which are originally used in animals can also suppress PCD in plants, indicating conserved mechanisms between animal and plant. But unlike the conserved sequences of PCD genes among different animal species, through the comparative study of plant and animal genomes, few conserved PCD genes were found, making the mechanism of PCD in plants a mystery. Hypersensitive response (HR), a typical form of plant PCD which prevents the spread of infection by pathogens, is analogous to the innate immune system in animals [5]. In both HR and necroptosis, oxidative burst by producing ROS is noticed and may be the major causes of downstream cell death execution. Thus the research of plant PCD will not only advance our understanding of how this process is regulated in plants, but also provide insights into animal PCD pathways, particularly mechanisms not existing in invertebrate model organisms.

**Figure 1:**
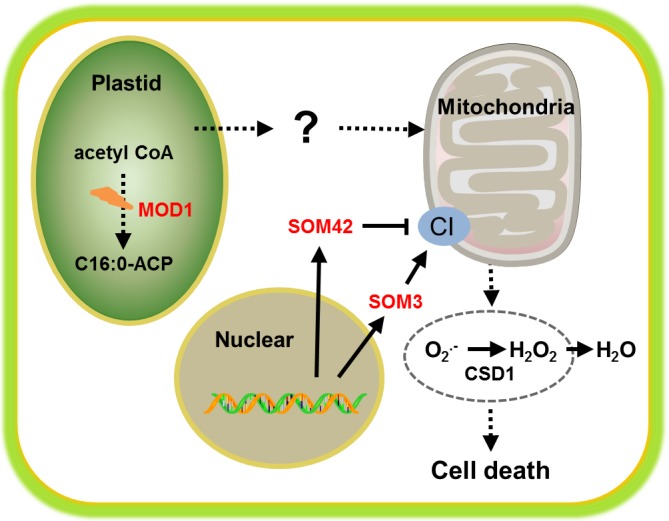
MOD1-mediated programmed cell death through mitochondrial complex I (CI)-generated ROS Knock out of SOM3, a component of CI, and overexpression of SOM42, a negative regulator of CI, can both repress cell death and ROS accumulation phenotypes of *mod1*. The red color highlights the mutant *mod1* and its supressors, *som3* and *som42*.

Our previous study showed that Arabidopsis mosaic death 1 (mod1), in which a recessive mutation causes the deficiency in fatty acid biosynthesis in plastids, displays pleiotropic phenotypes of typical PCD features [6]. Although MOD1 gene has been cloned for more than ten years, the relationship between fatty acid biosynthesis and programmed cell death remains largely unknown. Our current work, now published in Cell Research [7], has elucidated that the mitochondrial Complex I-generated ROS plays an indispensable role in MOD1-mediated PCD and is required for full HR and optimum disease resistance in Arabidopsis (Figure 1). Firstly, we examined the ROS level in mod1, and found that both H2O2 and O2- are highly accumulated. Unlike animals, multiple plant organelles/cellular compartments including chloroplast, mitochondrion, peroxisome, plasma membrane, cell wall and endoplasmic reticulum could produce ROS, and the chloroplast ROS has been regarded central to plant PCD [5]. Through T-DNA insertion mutagenesis, we screened out two mod1 suppressors, som3 and som42, which could rescue the cell death and ROS accumulation phenotypes of mod1. SOM3 encodes a subunit of the mitochondrial ETC complex I, and disruption of SOM3 directly reduces complex I levels. SOM42 is a nuclear-encoded pentatricopeptide repeat protein, belonging to a highly conserved family in higher plants. Our experiments showed that SOM42 is localized to mitochondria and that overexpression of SOM42 negatively regulates complex I activity by affecting the maturation of the mitochondrion-encoded complex I NAD transcripts. The role of complex I in PCD is further confirmed through the suppression of mod1 phenotypes by other complex I mutants, pharmaceutical inhibitors of the complex I-generated ROS, and over-expression of cytoplasmic copper/zinc superoxide dismutase. Subsequent analysis of disease resistance against the Pseudomonas syringae bacteria specified by NOD-Like Receptors showed that intact mitochondria are required for full HR and optimum disease resistance. Deficiencies in complex I will lead to attenuated HR and enhanced bacteria growth. These findings strongly indicate that ROS generated in the electron transport chain in mitochondria plays a key role in triggering plant PCD and immunity, suggesting a unifying theme between plant and animal innate immunities. However, the nature of ROS-inducing signal travels from plastids to mitochondria is still unknown (Figure 1). Based on these observations, the mod1 mutant likely maintains a constitutive HR active state induced by signals from plastids, and may serve as an ideal material for mutagenesis study to isolate key signaling elements in the upstream of oxidative burst, which consequentially triggers PCD. Although this PCD initiation pathway in plants originates from plastids, it may share common signals with vertebrates for communications between cytosol and mitochondrion and provide new knowledge to therapeutically manipulate PCD and disease treatment in humans.
